# Medians seek the corners, and other conjectures

**DOI:** 10.1186/1471-2105-13-S19-S5

**Published:** 2012-12-19

**Authors:** Maryam Haghighi, David Sankoff

**Affiliations:** 1 Department of Mathematics and Statistics, University of Ottawa, 585 King Edward Avenue, Ottawa, Canada K1N 6N5

## Abstract

**Background:**

Median construction is at the heart of several approaches to gene-order phylogeny. It has been observed that the solution to a median problem is generally not unique, and that alternate solutions may be quite different. Another concern has to do with a tendency for medians to fall on or near one of the three input orders, and hence to contain no information about the other two.

**Results:**

We conjecture that as gene orders become more random with respect to each other, and as the number of genes increases, the breakpoint median for circular unichromosomal genomes, in both the unsigned and signed cases, tends to approach one of the input genomes, the "corners" in terms of the distance normalized by the number of genes. Moreover, there are alternate solutions that approach each of the other inputs, so that the average distance between solutions is very large. We confirm these claims through simulations, and extend the results to medians of more than three genomes.

**Conclusions:**

This effect also introduces serious biases into the medians of less scrambled genomes. It prompts a reconsideration of the role of the median in gene order phylogeny. Fortunately, for triples of finite length genomes, a small proportion of the median solutions escape the tendency towards the corners, and these are relatively close to each other. This suggests that a focused search for these solutions, though they represent a decreasing minority as genome length increases, is a way out of the pathological tendency we have described.

## Background

The median problem, namely to construct the genome, the sum of whose distances from three given genomes is minimized, is of biological interest because it is at the heart of several approaches to phylogenetic inference based on gene order. It is also of computational interest since it represents one of the major axes of generalizations of simple pairwise gene order comparison, and most but not all versions are NP-hard [1].

One concern about the median problem, perhaps of more pertinence to applications than to theory, is that the solution is generally not unique, and that different solutions may be of considerable distance from each other (e.g., [2]). A second concern has to do with a tendency, if the three input gene orders are relatively highly rearranged with each other, for the median to fall on or near one of these input orders (e.g., [3]), rather than "in the middle", as might be more intuitively satisfying.

In this study, based on a series of simulations, we investigate the simplest median problems, that of unsigned genes under the breakpoint distance and that of signed genes under the breakpoint distance. We make use of a reduction of the problems into the Traveling Salesman Problem (TSP) [4], which we can now rapidly solve for genomes with thousands of genes [5]. We find that, indeed, as gene orders become more random with respect to each other, and as the number of genes increases, the median does indeed tend to approach an input genome, in terms of the distance normalized by the number of genes. Moreover, with the same input genomes, there are different solutions that approach each of the corners. We formalize these observations in terms of a conjecture.

We generalize this conjecture to the case of the median of four or more genomes. We also conjecture that the phenomenon of medians "seeking corners" carries over to other distances often applied to gene orders. Finally we discuss how it fits in with more general ideas of loss of evolutionary signal as gene orders become increasingly rearranged.

### The breakpoint median problem for circular chromosomes

For the unsigned case, we consider genomes modeled as (single) circular permutations on genes 1, …, *n*. Let *A *= *a*_1_, …,*a_n _*be such a permutation. The unordered pair (*a_i_*, *a_i_*+1) are called *adjacent*; they constitute an *adjacency *on *A*, for 1 ≤ *i *<*n*. In addition, circularity means that *a_n _* is adjacent to *a_1 _*.

Consider two unsigned genomes *A *= *a*_1_, ..., *a_n _*and *B *= *b*_1_, ..., *b_n _*on the same set of *n *genes. If two genes *g *and *h *are adjacent in *A *but not in *B *(that is, *gh *or *hg *do not appear in *B*), then they determine a *breakpoint*. The *breakpoint distance d*(*A*, *B*) between *A *and *B *is defined as the number of breakpoints in *A *(or, equivalently, in *B*). This can be calculated as *d*(*A*, *B*) = *n *− adj(*A*, *B*), where adj(*A*, *B*) is the number of adjacencies in common between *A *and *B*.

For a signed genome, each gene is assigned a positive or negative orientation. If gene *h*, with a given orientation in *A*, follows gene *g*, also with a given orientation, which we write *gh*, then if either *gh *or −*h *− *g *is in *B*, this constitutes a common adjacency in the two genomes. Otherwise the two genes determine a breakpoint.

Given three genomes *A, B*, and *C *on the same set of *n *genes, the breakpoint median problem is the problem of finding a genome *M*, called the *median*, such that *d*(*M*, *A*) + *d*(*M*, *B*)+ *d*(*M*, *C*), the *median sum*, i.e., the sum of the breakpoint distances between *M *and the given genome is minimized. This definition holds for both unsigned and signed genomes.

More generally, for *k *≥ 3, the *k*-median problem for breakpoints requires, for *k *given genomes *A*_1_, …, *A_k _*on the same set of *n *genes, finding a genome *M *such that the median sum $\sum_{i=1}^{k}\;d\left(M,Ai\right)$ is minimized. Where the meaning is clear, we will use the term "median" to refer to 3-medians.

The unichromosomal breakpoint median problems are known to be NP-hard ([6] and [7]), as are most, but not all, versions of the median problem, with metrics different from the breakpoint distance and/or on spaces of genomes different from that of circular unichromosomal genomes [1].

Nevertheless, by reducing the *k*-breakpoint median problem to the TSP [4], we can solve instances containing many thousands of genes rapidly [5], making use of *Concorde*, a software package that combines many of the recent advances in the field to rapidly produce TSP solutions [8].

Given *k *≥ 3 genomes *A*_1_, …, *A_k _*, to reduce the *k*-median problem for unsigned genomes to the TSP on *n *vertices, let *G *be a complete graph of the *n *vertices, where each vertex represents one gene. For each edge *xy *let *v*(*xy*) be equal to the number of times the genes corresponding to *x *and *y *are adjacent (do not form a breakpoint) in genomes *A*_1_, …, *A_k_*, so *v*(*xy*) can be any value among 0, …, *k*. Define the edge weight *w*(*xy*) = *k *− *v*(*xy*). Then a solution of the TSP on *G *with weights *w*(·), namely a minimum weight Hamilton cycle, defines a genome with a minimum sum of breakpoint distances to the *k *given genomes.

A similar strategy transforms the median problem for the signed genome problem to the TSP.

### The conjectures

We start with the unsigned case. For a given *n *≥ 1, consider a number of random genomes drawn independently from the set of all circular permutations, each with probability 2/(*n *− 1)!.

Let $P_{n}$ be the set of genomes containing *n *genes. For *A *∈ $P_{n}$, let the neighbourhood of *A *be

(1) $$N_{\varepsilon }\left(A\right)=\left\{B\in P_{n}|\frac{d\left(A,B\right)}{n}<\varepsilon \right\},$$

in other words, the set of genomes that are close to *A *in the normalized sense.

We note that for all *A, B *∈ $P_{n}$

(2) $$\frac{d\left(A,B\right)}{n}\leq 1,$$

because there can be no more than *n *breakpoints between two genomes of length *n*.

We impose a uniform measure *p_n _*on $P_{n}$, so that *p_n_*(*A*) = 1/(*n *− 1)! for all *A *∈ $P_{n}$. Then for random *A*, *B *∈ $P_{n}$, for large *n *the number of adjacencies approaches a Poisson distribution with parameter λ = 2 [9], so that

(3) $$\begin{array}{llll} E\left(\frac{d\left(A,B\right)}{n}\right) & \sim \frac{n-2}{n}\; & & \;\\ & \to 1\; & & \;\\ & \; & \end{array}$$

as *n *increases.

We propose the following:

**Conjecture 1 "Medians Seek the Corners" ***For any ε *> 0, δ > 0, *there is an n′*, *such that if A*_1_, …, *A_k _are k genomes drawn at random from *$P_{n}$, *where n *>*n′*, *and M is a k-median for these genomes, then*

(4) $$|p_{n}\left\{M\in N_{\varepsilon }\left(A_{i}\right)\right\}-1/k|<\delta ,$$

*for i *= 1, …, *k*.

It is important to note that not only would a median tend to be close to one of the input genomes *A*_1_, …, *A_k_*, but other median solutions for the same input genomes would simultaneously be close to each of the other input genomes, in equal proportions.

**Corollary 1 **For *n *= 1, …, *if A*_1_, …, *A_k _are k genomes drawn at random from *$P_{n}$, *then the expected normalized median sum*

(5) $$E\left(\frac{\sum_{i=1}^{k}d\left(M,A_{i}\right)}{n}\right)\to k-1,$$

*as n *→∞.

**Corollary 2 ***As n *→∞, *if A*_1_, …, *A_k _are k genomes drawn at random from *$P_{n}$*, and M*_1 _*and M*_2 _*are two medians of these k genomes, then*

(6) $$E\left(\frac{d\left(M_{1},M_{2}\right)}{n}\right)\to \frac{k-1}{k}.$$

We now turn to the case of signed genomes. Here, not only are there (*n *− 1)! gene orders, but there are 2^*n *^ways of assigning orientations to the genes. Thus the set $Q_{n}$ of all genomes contains 2*^n^*(*n *− 1)! elements. The definition of a neighborhood in Eq. (1) carries over with $P_{n}$ replaced by $Q_{n}$. For the uniform measure *q_n _*on $Q_{n}$, the Poisson parameter for the number of common adjacencies in two genomes is $\frac{1}{2}$ instead of 2 [9], but the limiting value of the normalized breakpoint distance is still 1, as in Eq. (3).

Then Conjecture 1 and Corollaries 1 and 2 are also proposed for the signed case, where $P_{n}$ replaced by $Q_{n}$ and *p_n _*is replaced by *q_n_*.

The conjecture, and its corollaries, might seem counterintuitive, especially if the median is conceived of as being "in the middle" of the input genomes. For example we could imagine constructing a genome containing a proportion 1/*k *of its adjacencies in common with each of the random input genomes. Its normalized distance would then be approximately (*k *− 1)/*k *from each of them, for a combined median sum of *k *− 1, the same as in the Corollary 1. Moreover, this would accord well with the notion of the median as being in the middle. However, such medians would not satisfy Corollary 2.

## Results

While awaiting formal proof of the conjecture, or its disproof, we can offer some observations based on simulations.

To generate a random genome we applied a series of rearrangements to the identity permutation 1, …, *n*. Though there are many alternative ways of gradually randomizing the genome, for convenience, our rearrangements all consisted of swapping the positions of two genes, chosen at random on the genome. This does not privilege any particular biological model for evolution, but simply represents a general way of gradually introducing random differences among the genomes.

For signed genomes, we also randomized the orientation of each swapped gene.

To get a sample of many alternative solutions to the median problem, we varied the seed used by Concorde to initialize its solution to the TSP. For our purposes it is desirable to sample uniformly from the entire set of medians for any one instance. Lacking an analysis of the internal workings of Concorde, we simply noted that the solutions seemed maximally diverse, as predicted by Corollary 2, and they showed symmetric tendencies with respect to the presentation order of the input genomes; i.e., there was no tendency for more genomes to be close to *A_i _*than to *A_j_*, for any *i*, *j *= 1, …, *k*.

**Figure 1:**
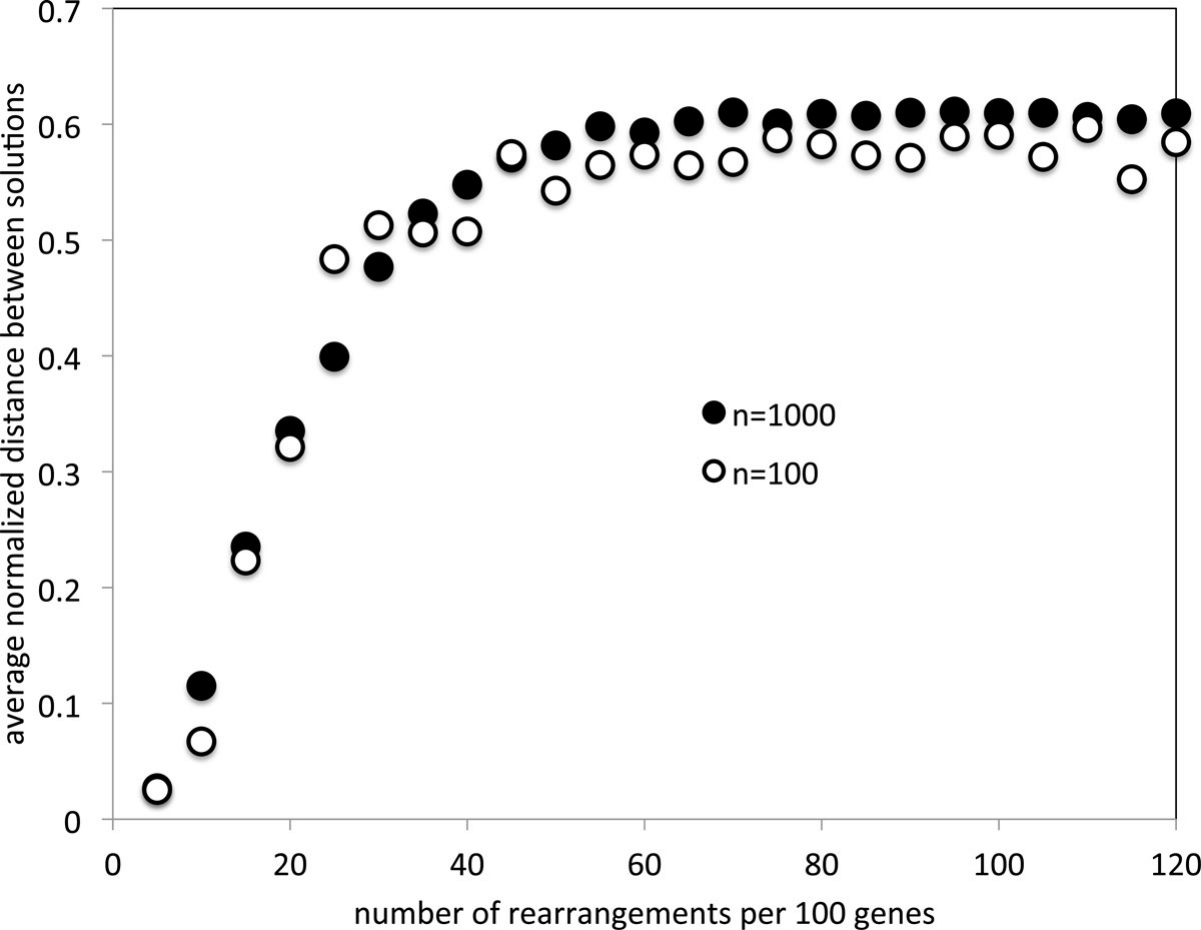
**Evolution of the average distance between median solutions as the input genomes become randomized**.

The first set of simulations for unsigned genomes depicted in Figure 1 shows how, for *k *= 3, the average normalized median sum increases at an identical rate (when not only the sum but also the number of rearrangements is normalized), for *n *= 100 and *n *= 1000, and approaches an asymptote of 2 at about 50 or 60 rearrangements per hundred genes. Of note is that the limiting value for *n *= 100 is slightly lower than that for *n *= 1000.

**Figure 2:**
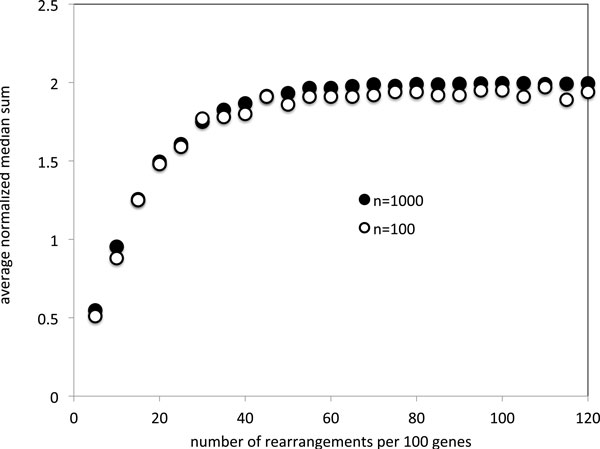
**Evolution of the median sum as the input genomes become randomized**.

For these same simulations, Figure 2 shows how the average distance between different solutions to the same instance of the median problem grows in the same way for *n *= 100 and *n *= 1000, and approaches an asymptote of 0.6 at about 50 or 60 rearrangements per hundred genes. Again, the limiting value for *n *= 100 is slightly lower than that for *n *= 1000, and both are considerable lower than the value of 2/3 predicted by Corollary 1; this observation will be understandable in the light of the results of the next section.

Simulations involving signed genomes gave very similar results to those depicted in Figures 1 and 2. The key analysis will be detailed in the next section.

## Medians at the middle

In the simulations, most of the solutions to the median problem were distributed evenly to the neighborhoods of the three input genomes. But a few were approximately equidistant from the the three of them: *d*(*M, A*_1_) ~ *d*(*M, A*_2_) ~ *d*(*M, A*_3_). This did not affect the median sum trends since, of course, as medians, these have the same sum as the ones near the input genomes. They do, however, affect the average distance between solutions, since they are closer together and, more important, closer to all of the input medians than the latter are to each other.

**Figure 3:**
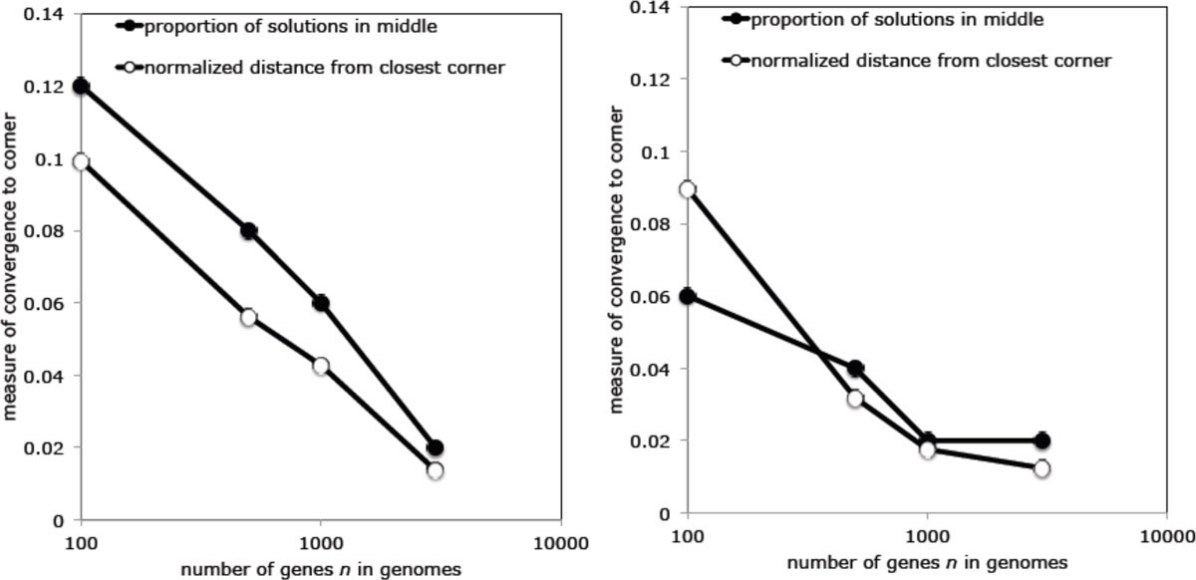
**Median drift towards the corners and transience of the middle solution**. Left: unsigned genomes. Right: signed genomes.

To further investigate the role of these "medians in the middle" we measured the average distance of median solutions from the closest input genome, and counted the number of centrally located medians out of 50 for each simulation. To ensure randomness, the inputs were generated with 300 random swaps (each swap involving up to four new breakpoints) per 100 genes in a genome, so that there will remain very few adjacencies in common with the identity permutation and, especially, with the other input genomes. The results are depicted in Figure 3 for both unsigned and signed median problems, where it can be seen that as n gets larger, the proportion of medians in the middle gets smaller and the average distance of medians from the nearest input genome drops at the same time.

In sum, while there are four types of median solution to each instance of the median problem with random input, three in the neighbourhoods of the input genomes, and one in the middle, the latter is of diminishing frequency; its measure goes to zero as *n *→∞.

## Generalization to higher *k*

**Figure 4:**
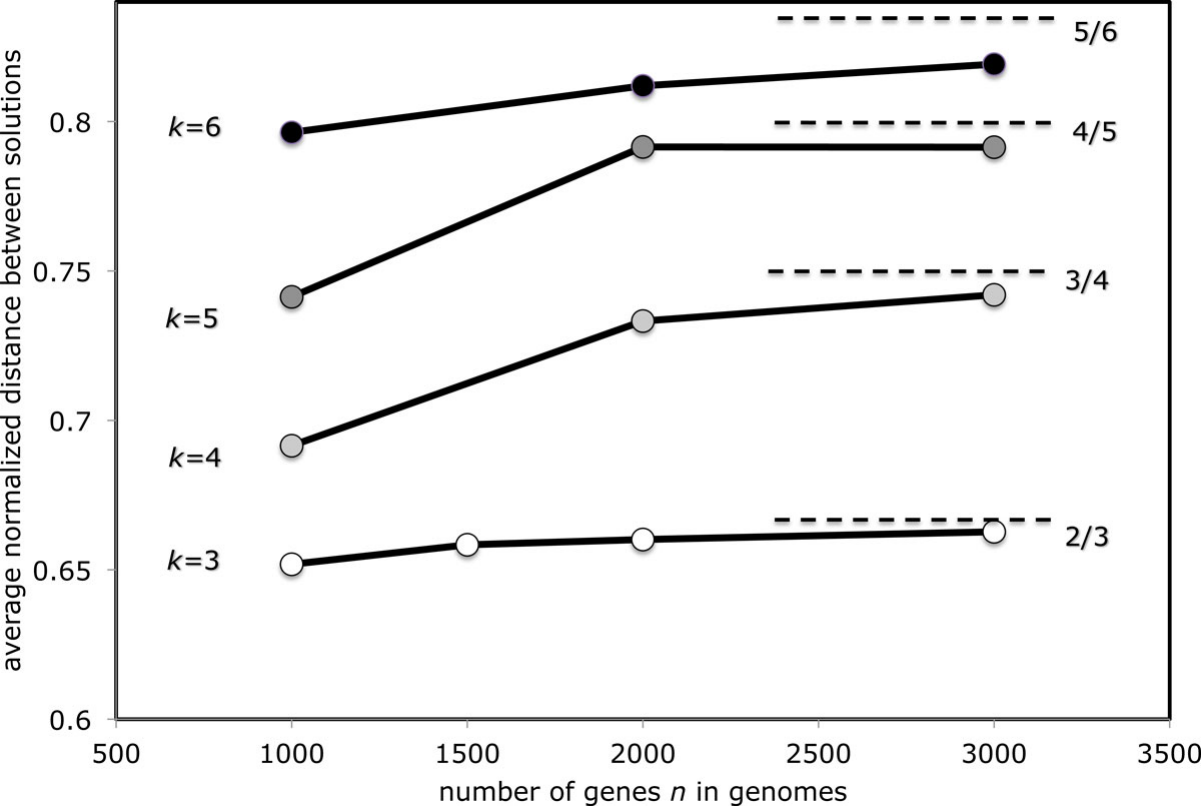
**Evolution of the average distance between median solutions as *k *input genomes become randomized**.

Simulations with *k *> 3 unsigned genomes confirm that our conjecture is valid beyond the usual 3-median case. Figure 4 shows that while convergence towards the asymptote (*k *− 1)/*k *slows as *k *increases, there is little doubt that this value is correct.

**Figure 5:**
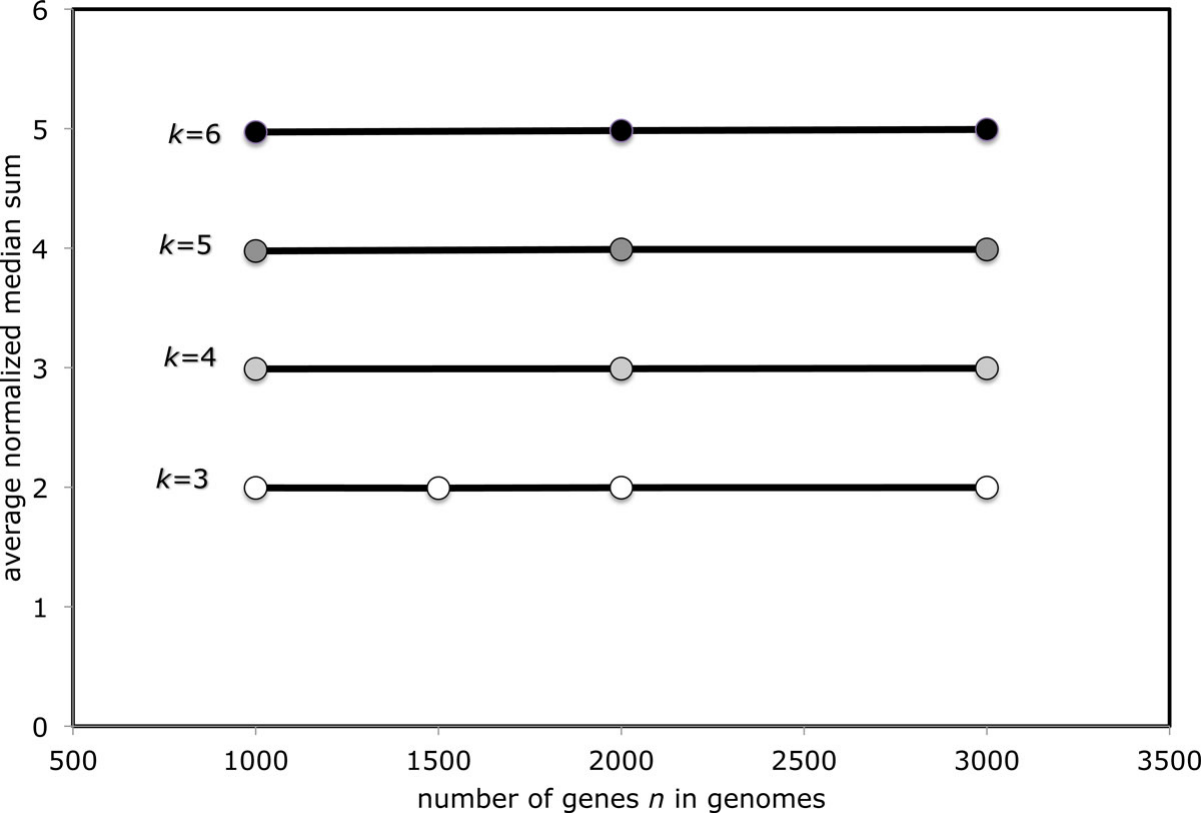
**Evolution of the median sum as *k *input genomes become randomized**.

Figure 5 again shows that the average normalized median sum converges to the asymptote *k *−1 predicted by Corollary 1, and this convergence is faster than that of the average distance between solutions.

Taken together, Figures 4 and 5 suggest that the set of medians in the middle has a greater measure and is slower to disappear as k becomes larger. Nevertheless, our ability to analyze genomes with thousands of genes enables us to affirm that the eventual trend towards "medians in the corners" holds for these *k *as well.

## Discussion

Although it would of course be good to have a proof of our conjecture and its corollaries, the simulations allow us a degree of confidence that they are true. There is a remote possibility that varying the seed used by Concorde does not lead to a uniform sample of median solutions, but this seems unlikely. One indication that there is no presentation-order artifact is that all three corners accumulate solutions to the same extent.

The solutions, of course, pertain only to random genomes. The gradual increase seen in Figure 2 may in part be due to a bonafide increase in the distances from a centrally located median to the corners. Nevertheless this increase in the median sum necessarily involves a component caused by the drift towards the input genomes, a component that dominates as the asymptote is approached. Furthermore, the increase seen in Figure 1 is more definitively suggestive of a set of three alternate solutions, each heading, with increased *n*, towards the input genomes in the corners.

These results imply that an unreflecting use of the median in comparing three even moderately scrambled genomes, and as the inner optimization step of a small phylogeny analysis, with ancestral gene reconstruction, is methodologically dangerous. A median at a corner contains no compromise information from the other two genomes. The tendency for the medians to seek a corner is a mathematical artifact of the notion of breakpoint or of some more general concept in the comparison of permutations, and should certainly not be attributed any biological significance.

**Figure 6:**
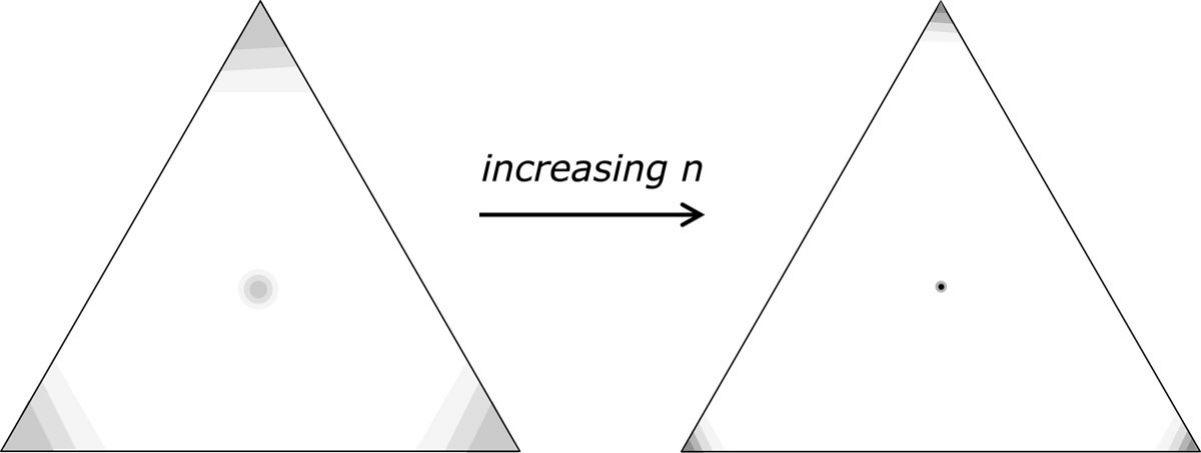
**Median solutions increasingly concentrated (shaded regions) around corners and shrinking at compromise positions**.

All is not lost, however! Recall that we have actually identified four median tendencies, not three. (Or *k *+1, not just *k*.) A minority of medians remain near the middle, and these definitely represent compromises among the three (or k) input genomes. Of course, these medians are rare, and become rarer as the inputs become longer and more random, as in Figure 6. Nevertheless, they exist, and are eminently interpretable biologically.

As a consequence, we suggest that applications of median methods should entail the comparison of many alternative medians, the identification and discarding of those contaminated by the drift towards the corner, and the search for the rare median that genuinely reflects a compromise among the input genomes. This may be done in an objective way since the set of medians will have four regions of high probability in the space of genomes, separated by large regions of low probability. Most of the probability will be concentrated on the neighborhoods of the input genomes. Finding the "poor cousin" in the middle may require the generation of large numbers of candidate solutions, but given the computing resources, this seems imperative if we want to make biological sense.

The computational difficulty traditionally ascribed to the median problem, especially when the input genomes are highly rearranged with respect to each other, would seem to preclude this approach. With breakpoint medians, however, computing time need not be a problem. Use of an efficient TSP solver allowed us to find medians when *n *= 3000, with maximally rearranged genomes, in seconds, and thus explore a fairly large sample of optimal solution space. Indeed, the limiting factor in our experimental set-up was memory, not time. Another advantage of breakpoints via the TSP approach is that it is not appreciably harder when the genomes are highly scrambled than when they are only moderately rearranged, or for larger *k *compared to *k *= 3.

Finally, we offer a further conjecture, which seems compelling to us, but for which we have only rough justification, and which moreover is unlikely to win many believers. We conjecture that breakpoint medians for the minimum reversals metric or the double-cut-and-join metric will also seek the corners as genomes become longer and more rearranged, although this effect may require relatively large *n *to become dominant. Furthermore, this should obtain for multichromosomal genomes as well, as long as the number of chromosomes (and hence chromosome ends) is bounded. This conjecture is motivated by the closeness with which these metrics follow the breakpoint metric when genomes are randomly generated [10], or have very high "breakpoint re-use" scores. Unfortunately it will be difficult to resolve this conjecture for rearrangement-based metrics using simulations. Using the best current methods [11], computing exact medians for genomes of size *n *≥ 100 under these metrics is computationally costly when the input genomes are even moderately scrambled.

## Authors' contributions

MH and DS did the research and wrote the paper.

## Competing interests

The authors declare that they have no competing interests.
